# Co-Occurring Hemolysis and Methemoglobinemia After COVID-19 Infection in Patient With G6PD Deficiency

**DOI:** 10.7759/cureus.35020

**Published:** 2023-02-15

**Authors:** Manar A Malakah, Bayan A Baghlaf, Samaher E Alsulami

**Affiliations:** 1 Internal Medicine, King Abdullah International Medical Research Center, Jeddah, SAU

**Keywords:** hypoxia, g6pd deficiency, hemolytic anemia, hemolysis, methemoglobinemia, covid-19

## Abstract

Hemolytic anemia and methemoglobinemia are known complications in patients with glucose-6-phosphate dehydrogenase (G6PD) deficiency. They can be elicited by various oxidative stressors.

Here we report a case of an adult with the first episode of G6PD deficiency associated hemolysis and methemoglobinemia after acquiring COVID-19 infection, who had no recent exposure to oxidative drugs or fava beans. A 52-year-old gentleman known to have myocardial bridging on aspirin and beta-blocker, with no other medical illnesses, developed anemia symptoms, jaundice, and hypoxia after contracting COVID-19 infection. Further laboratory work revealed non-immune hemolytic anemia, methemoglobinemia, and a positive G6PD screen test. He was treated conservatively with a blood transfusion, and his oxygen saturation improved thereafter.

With the widespread COVID-19 infection and its morbidity worldwide, it is crucial to consider methemoglobinemia in the differential diagnosis of hypoxia. Testing for G6PD is an essential next step in such cases, as starting methylene blue in G6PD deficiency can worsen hemolysis. Apart from COVID-19, there is no other identified trigger for the acute event in this patient. It is not known whether COVID-19 infection alone is enough to result in G6PD deficiency-associated hemolysis and methemoglobinemia simultaneously.

## Introduction

Glucose-6-phosphate dehydrogenase (G6PD) deficiency is an X-linked disease where there is an enzymatic defect that makes red blood cells prone to hemolysis after exposure to oxidative stress. It is the most common enzyme deficiency in humans worldwide [1]. G6PD is the rate-limiting enzyme in the pentose phosphate pathway (PPP), where nicotinamide adenine dinucleotide phosphate (NADP) is reduced to NADPH. NADPH is an essential component in many steps of normal hemostasis. It is essential for the red blood cells’ integrity against oxidative injuries. It also has an antimicrobial role in innate immune response, as it is needed in releasing antioxidants against infectious pathogens. Another function of NADPH is helping in the reduction of methemoglobin and restoring the normal function of hemoglobin [2-5].

Methemoglobin has a reduced ability to bind oxygen; therefore, it is essential to maintain methemoglobin levels within the normal range to ensure efficient oxygen delivery to the tissues [6]. Patients with G6PD are usually asymptomatic, and episodic intravascular hemolysis can develop after exposure to oxidative agents, fava beans, medications, or infections, where many free radicals accumulate and find no defense mechanism against them [5].

The global pandemic of COVID-19, with more than 600 million confirmed cases worldwide, is associated with varying severity of presentation and complications. Understanding of the nature of COVID-19 disease is still evolving [7-10]. There are a few reported cases of COVID-19 with G6PD deficiency who were found to have simultaneous hemolytic anemia and methemoglobinemia. Most of the cases were associated with the use of hydroxychloroquine [11-13], but only two cases found no clear trigger for the acute episode in adults [14,15]. A recently reported case of co-occurrence of hemolysis and methemoglobinemia in pediatric patients with COVID-19 in the absence of triggering medication [16].

## Case presentation

A 52-year-old male with a background of myocardial bridging on bisoprolol and aspirin, no previous interventions, and no other medical illnesses. He presented to a private hospital three days before his presentation to our hospital with a history of fever for one day. He was found to be hypoxic, his labs showed anemia hemoglobin of 7 g/dl, and COVID-19 polymerase chain reaction (PCR) was confirmed through a nasal swab. The patient was advised to go to a tertiary hospital for further management.

Upon presentation to our emergency department, his fever has resolved; however, he was complaining of dizziness and lightheadedness for three days, as well as a single episode of syncopal attack and dark urine. The patient denied any dyspnea or other respiratory symptoms. He had no recent sick contacts and no previous personal or family history of anemia or hemolysis. He was recently vaccinated by Pfizer for COVID-19 a week before the symptoms started. 

On examination, he was comfortable and not in respiratory distress; his oxygen saturation on the pulse oximeter was 85% on room air, picking up to only 88% with 15L oxygen through a non-rebreather face mask, blood pressure of 132/62 mmHg, heart rate of 68, respiratory rate of 20 and temperature of 37°C. He had scleral icterus; the remained physical examination was unremarkable. Initial investigations revealed normocytic anemia hemoglobin of 5 g/dl with elevated markers of hemolysis, Lactate dehydrogenase (LDH) of 1793 U/L, total bilirubin of 67 umol/L, direct bilirubin of only 16.6 umol/L and low haptoglobin of <0.058 g/L. COVID-19 PCR was repeated and found to be positive. Chest X-ray was unremarkable. An arterial blood gas (ABG) was done as there is a significant discrepancy between the patient’s clinical presentation and his oxygen saturation, and it revealed a partial pressure of oxygen (PaO2) of more than 99 mmHg, oxygen saturation (SaO2) of 99% with high methemoglobin level of 5.8% (0.0-1.5%). Additional hemolysis testing revealed a negative Coombs test and a positive G6PD screen (Table 1). Peripheral blood film showed polychromasia, and no bite cells or schistocytes were found.

**Table 1:** Laboratory test results during admission and follow up MCV = Mean Corpuscular Volume; MCHC = Mean Corpuscular Hemoglobin Concentration; ANC = Absolute Neutrophil Count; ALC = Absolute Lymphocytes Count; ALP = Alkaline Phosphatase; ALT = Alanine Aminotransferase; AST = Aspartate Aminotransferase; LDH = Lactate Dehydrogenase; CRP = C-Reactive Protein; PaO2 = Arterial Partial Pressure of Oxygen; PaCO2 = Arterial Partial Pressure of Carbon Dioxide; SaO2 = Arterial Oxygen Saturation; G6PD = Glucose-6-phosphate Dehydrogenase.

Labs	Day 1 upon admission	Day 3 upon discharge	Follow up after 3 months	Reference range
Hemoglobin	6.7	11	14.3	11.5-16.5 g/dl
Hematocrit	19.6	32	42.2	40-54%
MCV	97.4	89.8	93.3	76 – 96 fL
MCHC	34	33.8		32 – 36 g/dl
Reticulocyte (%)	2.93		2.44	0.2 – 2 %
White blood count	20.3	14	7.4	4 -11 x10^9/L
ANC	11.4	8.31	3.25	2 – 7.5 x10^9/L
ALC	5.26	3.41	3.21	1.5 – 4 x10^9/L
Platelets count	378	330	363	150 – 450 x10^9/L
Total bilirubin	67.3	4.1	12.7	2.1 – 15.5 umol/L.
Direct bilirubin	16		4.2	0 – 9 umol/L
ALP	68	61	57	39 – 114 U/L
ALT	21	25	20	6 – 28 U/L
AST	77	18	15	5 – 34 IU/L
LDH	1793	991	182	100 – 217 U/L
Haptoglobin	0.058			0.36 – 1.95 g/L
Creatinine	87	85	98	50 – 74 umol/L
Urea	6.9	6.5	6.8	1.9 – 5.7 mmol/L
CRP	78.2	7.7	5.9	0 – 5 mg/L
Ferritin	14640			4 – 102 ug/L
PaO2	99			83 – 108 mmHg
PaCO2	30			32 – 45 mmHg
SaO2	99			94 – 98 %
Methemoglobin	5.8			0.0 - 1.5 %
Vitamin B12	531			129 – 577 pmol/L.
Folic acid	21.4			8.4 – 38 nmol/L
Direct Antiglobulin Test	Negative			
G6PD screen	Positive			
G6PD level	0.10			4.6 – 13.5 unit/gm

The patient was admitted, received supplemental oxygen, and started supportive management with blood transfusion, oral folic acid, and ascorbic acid. The patient exhibited a remarkable improvement the next day, and he was discharged home after 48 hours with an uncomplicated clinical course. Repeated hemoglobin after two months was stable.

## Discussion

G6PD deficiency is a common cause of hemolytic anemia. The patient can present with episodic hemolysis after exposure to oxidative stressors, more often in children [1,3]. It is usually self-limiting, and most of the patients are treated conservatively by removing the precipitating agent and receiving blood transfusions [4,5].

Methemoglobinemia is a serious disease and an important differential diagnosis to consider in a patient with hypoxia detected on a pulse oximeter that is not corrected with supplemental oxygen. Patients with methemoglobinemia have various presentations; they may present with cyanosis, symptoms of anemia, dysrhythmias, neurological symptoms, coma, or death. The severity varies depending on the methemoglobin level and other multiple factors. Treatment should be initiated as soon as the disease is diagnosed. Treatment involves supplemental oxygen, removing oxidizing agents, methylene blue, and other modalities [6].

G6PD is crucial to provide NADPH, which has a role in both maintaining RBC integrity against oxidative agents and preventing high levels of methemoglobin. Oxidative agents include infections, the ingestion of fava beans, and specific medications [2].

The co-occurrence of hemolysis and methemoglobinemia in G6PD deficiency is uncommon. Few cases were stated in the literature reported co-occurrence of hemolysis and methemoglobinemia in G6PD deficiency secondary to a known inciting event due to hydroxychloroquine, rasburicase, fava beans ingestion, and secondary to acute infections [11-13,17,18]. A case reported described such an event following hepatitis E virus infection in a patient with G6PD deficiency [19]. Only three cases of acute hemolysis and methemoglobinemia were reported in G6PD-deficient patients with COVID-19 infection, and no other identified trigger [14-16]. Here, we report a new case of late-onset G6PD-associated hemolysis in an adult with concurrent methemoglobinemia after COVID-19 infection.

In our case, the patient had no identifiable oxidative agent, and the acute episode could be a consequence of an acute viral illness. It is still not clear whether COVID-19 infection is sufficient to cause G6PD deficiency-associated hemolysis and methemoglobinemia simultaneously. Our patient received the COVID-19 vaccine a week before this event. There are few reported cases of autoimmune hemolytic anemia following the COVID-19 vaccination [20-23]. However, there are no previously reported cases of G6PD deficiency hemolytic anemia or methemoglobinemia post-COVID-19 vaccine. It is not known if the COVID-19 vaccine has any role, yet it opens the floor for future studies. Our patient was asymptomatic apart from symptoms of hemolytic anemia, and he was not started on methylene blue, as it should not be given in cases of G6PD deficiency because of the risk of worsening hemolysis.

## Conclusions

Our case is reported to highlight the importance of considering methemoglobinemia in the differential diagnosis of hypoxia, particularly if it is not improving with supplemental oxygen. Although co-occurrence of hemolysis related to G6PD deficiency and methemoglobinemia is rare, its identification is critical, as giving methylene blue to G6PD deficient patients can worsen hemolysis.
